# Clinico-demographic factors associated with the treatment response to cognitive behavioral therapy for insomnia

**DOI:** 10.1186/s13030-024-00308-6

**Published:** 2024-05-17

**Authors:** Ayana Hotchi, Wataru Yamadera, Masayuki Iwashita, Tomohiro Utsumi, Misato Amagai, Sakumi Nakamura, Takako Suzuki, Masahiro Shigeta

**Affiliations:** 1 Department of Psychiatry, Jikei University School of Medicine, 3-19-18, Nishi- Shimbashi, Minato-ku, Tokyo, 105-8461 Japan; 2 Department of Psychiatry, The Jikei University Hospital, Minato-ku, Tokyo, Japan; 3 Department of Psychiatry, Jikei University Katsushika Medical Center, Katsushika-ku, Tokyo, Japan

**Keywords:** Insomnia, Chronic insomnia disorder, Cognitive behavioral therapy for insomnia, CBT-I

## Abstract

**Background:**

Cognitive behavioral therapy for insomnia (CBT-I) is among the recommended non-pharmacological treatments for patients with insomnia. While there are multiple reports on the effects of CBT-I treatment, few studies evaluating the factors associated with the treatment response to CBT-I have been reported. The present study aimed to confirm the effects of CBT-I in patients with insomnia and to examine the clinico-demographic factors that can predict the outcomes of CBT-I in these patients.

**Methods:**

Overall, 62 patients were included in the present study. To confirm the effectiveness of CBT-I, we compared the pre- and post-CBT-I therapy values of several sleep parameters. Furthermore, to identify the clinico-demographic factors that could be predictive of the treatment response to CBT-I, we performed generalized linear model (GLM) analysis.

**Results:**

The values of several sleep parameters were significantly lower after treatment than at baseline. The results of the GLM analysis revealed that sex and occupation were significantly associated with the treatment response to CBT-I.

**Conclusions:**

The present results suggest that several clinico-demographic factors should be considered in the treatment of patients with insomnia.

## Background

Pharmacological therapy is widely used for the treatment of insomnia, but the long-term use of hypnotics, especially gamma-aminobutyric acid-A receptor agonists (GABAA-RA), is known to be associated with a risk of regular dose dependence, daytime sleepiness, and impairment of psychomotor and cognitive functions [1]. Therefore, it would be desirable to develop effective non-pharmacological treatment methods for insomnia.

Under such circumstances, cognitive behavioral therapy (CBT), a psychotherapeutic technique, has attracted attention as a non-pharmacological therapeutic approach for patients with insomnia. Cognitive behavioral therapy for insomnia (CBT-I) is a proposed treatment method that works by modifying nonfunctional cognitive and behavioral patterns in such a way as to promote sleep. CBT-I consists of multiple components, including sleep hygiene education, cognitive therapy, relaxation techniques, stimulus control therapy, and sleep restriction therapy [2]. Numerous randomized controlled studies have demonstrated the beneficial effects of CBT-I in patients with insomnia [3–8], and CBT-I has recently been recommended as a first-line treatment for insomnia [9, 10].

Although it has been shown to be effective for treating insomnia, few studies have evaluated the factors that can influence the treatment response to CBT-I. As patients presenting with insomnia display a variety of characteristics, in the present study, we wanted to verify our hypothesis that the clinico-demographic characteristics of patients with insomnia can affect the treatment response to CBT-I.

Given this background, the present study aimed to: (1) confirm the effects of CBT-I on patients with insomnia, and (2) identify factors that can predict the effects of CBT-I on patients with insomnia.

## Methods

### Participants

The participants in the present study were outpatients who met the following inclusion criteria: (1) completed all sessions of CBT-I between March 2012 and April 2023 at the Department of Psychiatry, Jikei University Katsushika Medical Center, and (2) diagnosed by a psychiatrist as having chronic insomnia disorder based on the criteria of the International Classification of Sleep Disorders, Third Edition (ICSD-3) [11]. Patients who met any of the following exclusion criteria were excluded from the survey: (1) age less than 20 years; (2) difficulty in verbal communication due to severe physical or mental illness; and (3) physical difficulties in making regular hospital visits.

All participants had been started on treatment with hypnotic agents prior to the beginning of the study, and had expressed a desire to receive CBT-I for the treatment of insomnia. Of the comorbid diseases, psychiatric diseases were diagnosed according to the Diagnostic and Statistical Manual of Mental Disorders, Fifth Edition [12], and sleep disorders were diagnosed according to the ICSD-3.

To enable a reliable comparison of the data recorded before and after treatment, patients who had discontinued CBT-I before completing all sessions were excluded from the analysis.

### Measurements

The following characteristics of the patients were recorded at baseline: sex, age, type of insomnia (primary/comorbid), type of comorbid psychiatric disease/physical disease/sleep disorder, education level, presence/absence of employment, and doses of GABAA-RAs. The responses to the following questionnaires before and at 2 weeks after treatment completion were also analyzed: the Japanese version of the Insomnia Severity Index (ISI-J) [13, 14], the Japanese version of the Pittsburgh Sleep Quality Index (PSQI-J) [15, 16], and the Japanese version of the Zung Self-Rating Depression Scale (J-SDS) [17, 18].

The doses of GABAA-RAs were taken from the patients’ medical records at baseline and 2 weeks after completion of CBT-I. The data were analyzed quantitatively after converting the doses into flunitrazepam equivalents (1 mg = 1) [19]. In addition, whether hypnotics other than GABAA-RAs were taken before sleep was investigated.The 7-day mean values of the following parameters recorded in the participants’ sleep diaries during the weeks prior to the start and after completion of the last session of CBT-I served as the baseline and posttreatment data, respectively: (1) total sleep time (TST), (2) sleep efficiency (SE), (3) sleep onset latency (SOL), and (4) wake time after sleep onset (WASO).

### Treatment

CBT-I was implemented by two clinical psychologists who had received training from the Japanese Society of Sleep Research (JSSR) and who had experience in its implementation.

The therapists administered five sessions of CBT-I (face-to-face, biweekly sessions; 50 min/session) to each patient. The contents of the CBT-I conformed with the manual prepared by the JSSR [20], including interviews with the patients on the following topics: (1) knowledge about treatments for insomnia (sleep hygiene education), (2) relaxation (progressive muscle relaxation), (3, 4) sleep scheduling (sleep restriction therapy and stimulus control therapy), and (5) review and summary of past sessions. The content of each session was set as homework to check the degree of patient practice and was reviewed at the subsequent session. Each patient was instructed to maintain a sleep diary from 2 weeks before the start of the CBT-I sessions. Sleep scheduling was continued from the third to the last session. During the CBT-I period, each patient was examined by a physician once every 4 weeks. The physician-in-charge provided the usual treatment and did not discuss the contents of the CBT-I instructions with the patient. Guidance regarding reductions in the doses of the hypnotic drugs used was also given by the physician-in-charge in accordance with the relevant guidelines [21], as usually performed during clinical practice.

### Statistical analysis

IBM SPSS 24 for Windows (SPSS Japan Inc., Tokyo, Japan) was used for all statistical analyses. First, Wilcoxon’s signed-rank test was used to compare changes in the values of the sleep parameters recorded at baseline and post-CBT-I to confirm the treatment response. Next, we performed both univariate and multivariate generalized linear model (GLM) analysis to examine factors that influenced the response to CBT-I in patients with insomnia. Changes in ISI-J scores recorded at baseline and the posttreatment assessment were regarded as dependent variables. Univariate analysis was used to evaluate each predictor variable independently for its association with the outcome, while multivariate analysis simultaneously assessed all predictor variables for their combined effects. The analysis was conducted using the patients’ characteristics (age, sex, primary/comorbid insomnia, education level, duration of insomnia, employment status, PSQI-J score, J-SDS score, TST, SOL, WASO, SE, and dose of GABAA-RA) as covariates. The assumption was made that the response variable followed a normal distribution. In cases of significant findings in the GLM analysis, subgroup analyses were performed to explore associations, using chi-square or Mann-Whitney *U* tests as appropriate. For all tests, the alpha level was set to 0.05.

## Results

From among the 75 patients initially enrolled in this study, the data of 62 individuals who completed the planned treatment was available for analysis. Of the remaining 13 patients, eight discontinued the intervention and five were excluded because of incomplete questionnaire responses. The characteristics of the discontinuation group (*n* = 8) are summarized as follows: age (mean ± standard deviation): 57.4 ± 14.6;sex: male (M) = 3; employment status: employed = 5; insomnia type: primary = 6; education (years): 13.7 ± 2.9; and duration of insomnia (months): 66.9 ± 60.9. The comparison of background factors of the discontinuation and completion groups was done using the chi-square and Mann-Whitney *U* tests. The results revealed no statistically significant differences in age, sex, employment status, insomnia type, education level, or duration of insomnia (*p* > 0.05 for all variables). The reasons for withdrawal were personal reason (*n* = 5), deterioration of physical condition (*n* = 1), and unknown (*n* = 2).

### Baseline characteristics of the patients with insomnia

The characteristics of the patients with insomnia at baseline are shown in Table 1.

**Table 1:** Baseline characteristics

Age (years)	63.1 ± 14.8
Sex: male/female	27/35
Occupational status: employed/unemployed	25/37
Type of insomnia: primary/comorbid	34/28
Education (years)	13.6 ± 2.2
Duration of insomnia (months)	109.5 ± 112.9

Values are expressed as mean ± standard deviation or number

### Comparison of sleep parameters recorded at baseline and after CBT-I

A comparison of the sleep parameters recorded at baseline and after CBT-I is shown in Table 2. The ISI-J score, PSQI-J score, SOL, WASO, and GABAA-RA dose after CBT-I were significantly lower than the corresponding values at baseline. The SE after CBT-I was significantly higher than that at baseline. No significant differences were found in the other variables examined. Regarding the usage of hypnotics other than GABAA-RAs, the study observed suvorexant use in seven cases, lemborexant use in five cases, and ramelteon use in none. The dosages of these medications after completing CBT-I either remained consistent with the pre-CBT-I levels or were discontinued.

**Table 2:** Comparison of the sleep parameters recorded at baseline and after CBT-I

	Baseline	Posttreatment	
	(mean ± SD)	(mean ± SD)	p value
ISI-J score	16.2 ± 5.0	10.5 ± 5.9	< 0.01**
PSQI-J score	9.8 ± 3.6	7.3 ± 3.1	< 0.01**
J-SDS score	42.4 ± 8.4	41.1 ± 8.9	0.23
Total sleep time (min)	347.2 ± 86.5	362.8 ± 77.9	0.08
Sleep onset latency (min)	42.6 ± 33.7	30.6 ± 27.4	< 0.01**
Wake time after sleep onset (min)	31.2 ± 32.1	17.5 ± 24.1	0.01*
Sleep efficiency (%)	73.0 ± 13.4	82.0 ± 14.0	< 0.01**
Dose of GABAA-RA	1.10 ± 0.74	0.84 ± 0.65	< 0.01**

CBT-I, cognitive behavioral therapy for insomnia; SD, standard deviation; ISI-J, Japanese version of the Insomnia Severity Index; PSQI-J, Japanese version of the Pittsburgh Sleep Quality Index; J-SDS, Japanese version of the Self-Rating Depression Scale; GABAA-RA, gamma-aminobutyric acid-A receptor agonist

Wilcoxon signed-rank test: *p < 0.05, **p < 0.01

### Results of the GLM analysis

The results of the GLM analysis are detailed in Table 3. Univariate analysis revealed female sex and unemployed status as being significantly associated with the reduction of the ISI-J scores after CBT-I. Conversely, multivariate analysis showed significant associations with older age, female sex, primary type of insomnia, shorter duration of insomnia, and unemployed status. When considering both univariate and multivariate results together, it becomes evident that female sex and unemployed status consistently emerge as significant predictors of treatment response to CBT-I in patients with insomnia.

**Table 3:** Results of generalized linear model analysis to identify the patient characteristics significantly associated with improved scores on the ISI-J

	Unadjusted				Adjusted			
Variables	β	95% CI		*p value*	β	95% CI		*p value*
		Lower	Upper			Lower	Upper	
Age	0.053	-0.055	0.162	0.33	0.119	0.026	0.211	0.01*
Sex (female)	5.494	2.555	8.432	< 0.01**	6.014	3.573	8.454	< 0.01**
Type of insomnia(primary)	4.148	1.047	7.248	0.09	3.961	1.419	6.502	0.02*
Education level (years)	0.149	-0.598	0.896	0.7	-0.206	-0.792	0.379	0.49
Duration of insomnia (months)	-0.009	-0.023	0.005	0.19	-0.011	-0.022	< 0.001	0.04*
Employment status (unemployed)	3.989	0.873	7.105	0.01*	5.096	2.118	8.073	< 0.01**
PSQI-J score at baseline	-0.079	-0.515	0.358	0.72	-0.065	-0.505	0.375	0.77
J-SDS score at baseline	-0.155	-0.357	0.046	0.13	-0.096	-0.248	0.057	0.22
Total sleep time (min)	0.002	-0.017	0.021	0.83	-0.008	-0.030	0.014	0.48
Sleep onset latency (min)	0.002	-0.049	0.053	0.94	0.029	-0.024	0.083	0.28
Wake time after sleep onset (min)	0.016	-0.046	0.079	0.61	-0.025	-0.074	0.024	0.32
Sleep efficiency (%)	-0.006	-0.126	0.113	0.92	0.031	-0.145	0.207	0.73
dose of GABAA-RA	0.145	-2.124	2.414	0.9	-1.687	-3.335	-0.039	0.05

ISI-J, Japanese version of the Insomnia Severity Index; PSQI-J, Japanese version of the Pittsburgh Sleep Quality Index; J-SDS, Japanese version of the Self-Rating Depression Scale; GABAA-RA, gamma-aminobutyric acid-A receptor agonist

*p < 0.05, **p < 0.01

### Results of the sex-specific analysis of ISI score changes based on employment status

We conducted additional analyses to investigate sex-specific outcomes related to employment status and changes in ISI scores before and after CBT-I. As outlined in Table 4, Mann-Whitney *U* tests revealed a significant difference in ISI score changes between employed and unemployed men, with unemployed men showing a significantly smaller change in ISI scores compared to employed men. No significant differences were observed among women based on employment status.

**Table 4:** Analysis of ISI Score Changes based on Employment Status, by sex

	Employed Men	Unemployed Men	*p*-value (Men)
ISI Score Changes (mean ± SD)	0.17 ± 4.76	-4.87 ± 4.87	0.016*
	Employed Women	Unemployed Women	p-value (Women)
ISI Score Changes (mean ± SD)	-6.77 ± 3.09	-8.64 ± 6.09	0.319

Mann-Whitney *U* test: *p < 0.05

## Discussion

### Summary of findings

In the present study, we confirmed significant improvements in sleep disturbance after CBT-I as assessed by the ISI-J and PSQI, as well as improvements in SOL, WASO, and SE.

Furthermore, our GLM analysis identified the following patient characteristics as being significantly associated with improved scores on the ISI-J subjective sleep questionnaire after CBT-I: female sex and unemployed status.

### Comparison with previous studies

Multiple meta-analyses have shown that CBT-I is associated with improvements in various sleep parameters and scores on sleep rating scales [3–8]. Consistent with these reports, our study also found improvements in SOL, WASO, and SE, as well as a reduction of the dose of GABAA-RA after CBT-I.

Few studies have examined differences in the effects of CBT-I by sex, and most of these have indicated the absence of any significant influence of sex on treatment outcomes [22–24]. Our analyses indicated that women are more likely to respond to CBT-I, inconsistent with previous reports. Furthermore, we found that unemployed status was predictive of better treatment responses to CBT-I among patients with insomnia.

#### Relation of sex and employment status to the effectiveness of CBT-I

In our cohort, we identified several clinico-demographic factors that significantly influenced the response to CBT-I among patients with insomnia. CBT-I treatment was significantly more effective among unemployed compared with employed patients. The work environment of each individual, especially the characteristics of the employment system of Japan, may explain this result.

The employment status of men and women in Japan differs substantially, with an employment rate of 83.9% for men and 71.3% for women (2021), a lower proportion of women in management positions (13.2%, 2021), and a higher proportion of women among short-time workers (15.0% for men and 39.0% for women, 2021) [25]. Another significantly different feature of Japan compared with other countries is the significant drop in the ratio of women participating in the labor force from their late 20s to 30s [25]. This reflects the tendency for women to quit their jobs for childcare after marriage and childbirth, and then return to the labor market thereafter.

Furthermore, the percentage of workers in non-regular employment has remained high in Japan for a long time. With companies seeking to reduce costs and adopt more flexible forms of employment, this has resulted in unstable employment and disparities in working conditions. In 2022, the percentage of non-regular workers in Japan was 53.4% for women and 22.2% for men, and increased with increasing worker age [26]. Such a social environment has led to a relatively high number of female full-time homemakers in Japan, potentially influencing our study results.

While our initial analysis suggested that women, with a lower overall employment rate, exhibited better outcomes with CBT-I, additional analyses revealed a more complex picture. Analyzing employment status within each sex revealed a significant treatment response difference among men, with employed men showing less improvement compared to non-employed men.

It is conceivable that being unemployed is relatively more likely to change daytime activity schedules and lifestyles, including behavioral changes during the day and before bedtime. These factors could explain the more beneficial outcomes of the sleep scheduling and hygiene components of CBT-I among the non-employed. Furthermore, insomnia has been reported to be associated with job stress and interpersonal conflicts in the workplace [27, 28]. Psychosocial factors associated with workloads and relationships in the workplace could also have influenced the treatment outcomes of CBT-I, particularly in the men in our study.

Our findings, revealing diverse treatment responses among employed and non-employed men, emphasize the interplay between sex, employment status, and treatment outcomes. Further research is essential to explore underlying factors and address potential confounding effects through thorough multivariate analysis.

Moreover, while we categorized participants into employed and unemployed groups in this study, acknowledging the diversity within employment structures, including part-time or full-time work, is crucial. Future studies should explore these differences to better comprehend how employment dynamics relate to insomnia outcomes.

### Interventions for patients with poor prognostic factors

In the present study, we found that male gender and positive employment status were predictive of poorer responses to CBT-I among patients with insomnia. Therefore, we believe that additional approaches may be needed for these patients. Patients with such psychosocial factors may require intensive psychosocial considerations. Given the limited effectiveness of CBT-I observed in specific groups, a re-evaluation of CBT-I components may be warranted. For instance, allocating more time to relaxation-focused approaches rather than adhering to sleep scheduling methods or incorporating mindfulness-based stress reduction could be a viable strategy for enhancing treatment outcomes in individuals identified as having poor prognostic factors. Other non-pharmacological treatments, light therapy, and other pharmacological treatments could be considered as additional or alternative treatments for these patients.

Looking beyond CBT-I, addressing an individual’s work situation is crucial. While delivering CBT-I directly to individuals remains beneficial, it is equally important to raise awareness at the societal level. Recognizing the importance of sleep and its susceptibility to work environments, promoting awareness through workplace improvements, and providing education on sleep hygiene on the societal level are therefore essential. This approach not only aims to enhance individual responses to insomnia interventions but also strives to build a broader understanding of the interplay between work environments and sleep health.

### Limitations

The present study has some limitations. First, the sample size was comparatively small, potentially impacting the generalizability of our findings and limiting the thorough evaluation of confounding factors. Second, the mean age of the participants was comparatively high, which may limit the broader applicability of the results. Third, as the present study was performed at a single institution, there are concerns regarding its validity. Further studies with larger, diverse samples and multi-center designs are needed to validate our findings and address these limitations.

## Conclusion

In the present study, we found that female sex and unemployed status were associated with better outcomes after CBT-I. These results suggest that certain clinic-demographic factors should be considered in the treatment of patients with insomnia. However, further investigations are needed to improve understanding and treatment strategies, especially for patients with poor prognostic factors.

## Data Availability

The datasets used and/or analyzed during the current study are available from the corresponding author on reasonable request.

## Abbreviations

CBT: Cognitive behavioral therapy
CBT-I: Cognitive behavioral therapy for insomnia
GABAA-RA: Gamma-aminobutyric acid-A receptor agonist
GLM: Generalized linear model
ICSD-3: International Classification of Sleep Disorders, Third Edition
ISI-J: Japanese version of the Insomnia Severity Index
J-SDS: Japanese version of the Zung Self-Rating Depression Scale
JSSR: Japanese Society of Sleep Research
PSQI-J: Japanese version of the Pittsburgh Sleep Quality Index
SE: Sleep efficiency
SOL: Sleep onset latency
TST: Total sleep time
WASO: Wake after sleep onset
